# Developing kind emotions in caring environments

**DOI:** 10.1098/rstb.2025.0141

**Published:** 2026-09-24

**Authors:** Ruth Speidel, Tal Orlitsky, Marian S. Schulz, Tina Malti

**Affiliations:** ^1^ Centre for Child Development, Mental Health, and Policy, University of Toronto Mississauga, Mississauga, Ontario, Canada; ^2^ Psychological and Brain Sciences, University of Toronto Mississauga, Mississauga, Ontario, Canada; ^3^ Humboldt Science Centre for Child Development, Leipzig University, Leipzig, SN, Germany

**Keywords:** kind emotions, empathy, caring relationships, prosociality, web of care, flourishing

## Abstract

Kind emotions (e.g. empathy for others, self-compassion and gratitude) motivate kind actions and involve understanding, appreciating or ‘feeling with’ both one's own and others' emotions. They are an essential capacity for humans' potential to care for fellow humans and non-human entities. How do such emotions evolve, and what type of caring environments and mechanisms are needed for their development? In this review, we provide insights into how children's kind emotions emerge, develop and are nurtured by caring environments. We begin by reviewing the development of kind emotions across the early years and identify consequences of kind emotions on prosociality and well-being. Next, we summarize research on the mechanisms and functions of caring environments in shaping the development of kind emotions, including caring relationships and practices of care across diverse relationships (e.g. with parents and peers) and layers of the ecosystem (e.g. familial, educational, societal and natural). Finally, we provide recommendations for future developmental research in this area.

This article is part of the theme issue ‘Mechanisms, development, phylogeny and functions of emotional expressions’.

## 1. Introduction

In a world filled with conflict, cruelty and violence, kindness and caring may seem hard to find. Yet, aggression and prosociality are both fundamental aspects of the human experience, and acts of kindness can uplift and inspire us in everyday life. The development of kind, prosocial individuals can provide a psychological foundation for humanity to survive and thrive [1]. Similarly, the intentional creation of caring environments can support individual prosocial orientations, health and well-being, and the formation of kinder communities [2].

Conceptually, kindness overlaps with prosociality, but they are distinct constructs. Prosociality refers broadly to the ways that individuals act to benefit others [3,4]. The motivations for prosociality can vary from other-oriented concerns such as empathy and compassion to more neutral motives like a desire to follow social norms, to more self-oriented concerns such as a desire for recognition, rewards or reciprocity [5]. In this paper, we focus on a narrower form of prosociality, such as kindness, which is a virtue that is characterized by a genuine concern for others' (and our own) well-being, and which is typically motivated by other-oriented factors such as empathy, compassion and ethical principles [6]. Kindness is a positive value across cultures and can be expressed through non-verbal means including emotions, thoughts and actions directed towards others or oneself [6]. In this review, we use the terms *kindness* and *caring* interchangeably as they are deeply interconnected. While the importance of kindness for well-being, social cohesion and peaceful human–nature relationships has been acknowledged, less is known about its developmental course and the caring environmental arrangements that are needed to nurture it. As such, we lack a clear understanding of how dimensions of kindness emerge and change across development, and how they are nurtured by caring environments. In this selective review, we define kind emotions and provide a conceptual framework for the study of the development of kindness in caring environments. We then describe the development of non-verbal expressions of kind emotions, followed by an examination of the role of different caring environments as mechanisms that facilitate kind emotions in childhood and adolescence. Lastly, we draw conclusions for future research and research-informed practices to nurture kindness and caring.

## 2. What are kind emotions?

We focus on kind emotions because they have been conceptualized as core elements and motivators of kindness [6]. As one theoretical anchor to frame our discussion of kind emotions, we use the *3Es* of social-emotional development, which are considered foundational for prosociality: emotion regulation, empathy for others and empathy for the self [7]. *Emotion regulation* is the capacity to manage and adjust the expression, occurrence and intensity of emotions [8]. Although emotion regulation is not a focus in this review, we acknowledge that it is intertwined with children's kind emotions across development. Indeed, emotion regulation supports children's capacity to manage empathy-related distress, which, in turn, supports their prosocial actions [8,9]. Meta-analytic evidence also shows links between better emotion regulation and higher empathy across the lifespan [10,11]. *Empathy for others* is the capacity to feel with others and includes an emotional component (i.e. the ability to feel the same emotion as another person) and a cognitive component (i.e. the ability to understand the thoughts and perspectives underlying another's emotion [7]). Three core kind emotions conceptualized within empathy for others are empathy (experiencing the same emotion as another), sympathy (feelings of concern for another) and compassion for others (feelings of concern accompanied by a motivation to help or reduce distress) [6]. Although these constructs are conceptually distinct (particularly in that sympathy and compassion more consistently motivate prosocial behaviour than empathy [12–14]), in this paper, we treat the three as components of the broader construct of ‘empathy for others’ given their other-oriented focus and that all have been linked to kindness and prosocial behaviour [6]. *Empathy for the self* encapsulates how children view themselves and includes an emotional component (i.e. self-compassion) and cognitive component (i.e. self-reflection and self-awareness) [6,7]. In this review, we focus on self-compassion as a key kind emotion because it parallels empathy for others while maintaining a self-oriented, relational focus. *Gratitude* is an additional kind emotion considered in this review. Gratitude is a positively valenced moral emotion that is experienced when one recognizes and feels appreciation for a benefit that has been provided to them [15]. Experiences of receiving kindness and care often elicit gratitude, and in turn, gratitude can function to motivate future prosocial and kind behaviour [16]. In this way, feelings of gratitude reflect and reinforce kindness.

Kind emotions develop together and impact each other throughout the early years and across the lifespan, and they are often conveyed through non-verbal expressions, including facial expressions, gestures, vocalizations, posture and prosocial behaviours [2,17]. These non-verbal signals serve functional roles in communicating children's affective state and supporting social interactions, including eliciting caring responses, signalling connection and care towards others and reinforcing prosocial norms of behaviour [1,6,17]. Understanding the development of kind emotions requires a focus on how these emotions are produced and expressed, and the functions they serve across childhood and adolescence. In childhood, expressions of empathy for others, self-compassion and gratitude may include facial expressions (e.g. concerned looks and smiling), gestures (e.g. approach behaviours, helping motions and self-soothing behaviours) and vocalizations (e.g. sighs of relief and pleasure) [17,18]. Notably, kind emotions like self-compassion and gratitude may lack more overt facial or vocal cues but are commonly expressed through touch and behavioural tendencies (e.g. self-soothing behaviours, approach behaviours and hugging) [17].

In this paper, we focus on empathy for others, empathy for the self (i.e. self-compassion) and gratitude because we conceptualize them as *kind emotions* that motivate kindness and that reflect underlying principles of compassion and gentleness towards others and oneself [6]. Kind emotions are considered foundational for various positive developmental pathways and have been shown to predict later increases in prosociality and decreases in aggression. Although the majority of research has most extensively focused on empathy for others [19–21], self-compassion [22] and gratitude [16,23] have also been empirically linked to children's prosocial behaviour, highlighting that they each function as motivators of prosocial behaviour.

Notably, although much of the research highlights a protective function of kind emotions, some research contends that excessive empathy for others, as well as altruism at the expense of the self, reflecting high empathy for others paired with low empathy for the self (i.e. costly altruism), may contribute to difficulties, including internalizing problems such as anxiety and depression [24,25]. In such cases, empathy for others may lead to disproportionate feelings of personal distress (i.e. self-oriented, unpleasant emotional reactions to another's distress) and result in focusing one's attention on reducing one's own experience of discomfort, interfering with the capacity to engage in prosocial responses towards another in need [21]. This suggests that a healthy balance of empathy for others, empathy for the self and gratitude may be optimal, as excessive levels of any may be dysfunctional and result in mental health issues.

## 3. The development of kind emotions from the early years to adolescence

### (a) Development of empathy for others

The capacities underlying empathy for others develop rapidly across the early years. What is less clear is the timing of their emergence. Growing empirical evidence from recent years indicates that empathy develops as early as infancy [26–29], although some researchers argue that empathy does not materialize until between 18 and 24 months [30]. Amidst these differing views, most scholars agree that the emotional and cognitive components of empathy for others unfold along differing timelines, with the emotional component emerging earlier and the cognitive component coming online later.

The emotional component of empathy (i.e. the ability to share another's emotional state) is considered an evolutionarily adaptive process, reflected in vicarious emotional arousal that emerges in infancy [31–33]. As early as one to two days after birth, newborns cry in response to another infant's cry [34]. This phenomenon has been observed consistently at one, three, six and nine months of age [35]. This emotional vicarious arousal is thought to gradually give rise to other-oriented responses based on three components: (i) cognitive understanding, which can rely on automatic, implicit processes or explicit, higher-order cognitive functions; (ii) self–other distinction, which may similarly operate in implicit or explicit forms; and (iii) the ability to regulate emotional arousal [36]. Indeed, studies have shown that as early as three months, infants display empathic concern across different tasks and over time, responding to others' distress through vocal, facial, gestural and postural behaviours [27]. Similar patterns have been observed in infants aged eight and nine months [26,28].

As infants progress into toddlerhood and early childhood, the cognitive component of empathy develops rapidly, whereby children become increasingly able to recognize and interpret others' emotions and the perspectives and intentions that underlie them [37]. Given that these capacities require more advanced cognitive abilities (i.e. perspective taking and the ability to distinguish between one's own and others' emotions), cognitive empathy develops later than emotional empathy [20,38]. In toddlerhood, children begin to engage in more complex, other-oriented responses to others in distress. Between 18 and 25 months, toddlers show more complex forms of empathic concern and are more likely to help, share or cooperate with someone who has been harmed than someone who has not, even when overt emotional cues are minimal, suggesting a developing ability to engage in perspective taking and internalized moral norms as well as stronger following of external norms, rather than relying solely on others' observable distress signals [39]. Evidence for increasing complexity in prosocial responses comes from studies using cross-sectional age comparison designs within toddlerhood. For example, when comparing prosocial behaviour in 18- and 30-month-olds, younger toddlers engage primarily in instrumental helping (i.e. assisting others in achieving action-based goals), whereas empathic helping (i.e. responding aimed at alleviating another's emotional distress) is less frequent and requires more communicative support from adults. By contrast, 30-month-olds help more readily, particularly in empathic contexts, and do so with less explicit guidance, suggesting a growing capacity to infer others' needs without explicit instruction [40]. These findings suggest that across toddlerhood, children's prosocial behaviour becomes increasingly differentiated and other-oriented, consistent with the development of cognitive empathy. However, across toddlerhood, children are more likely to help when the action is not costly to themselves [26].

By around the age of 4 years, children are able to understand and verbalize others' perspectives and understand that others may hold views different from their own [41]. Supported by these growing cognitive capacities, moving into middle childhood and adolescence, longitudinal evidence suggests that from ages 6 to 16 years, children become able to recognize increasingly complex emotions in others [42]. In parallel with these changes, helping behaviours increase and grow increasingly sophisticated in early and middle childhood [43,44]. Longitudinal research shows that empathy generally stabilizes in adolescence; in one study, 14–17-year-old adolescents' empathic concern was stable over time [45].

Overall, the findings suggest that empathy for others emerges early through emotional arousal, expands with the growth of cognitive capacities and eventually stabilizes into more complex and consistent forms of empathy.

### (b) Development of empathy for the self

Many of the same emotional and cognitive components that support empathy for others (e.g. self-reflection, self–other distinction and self-regulation) are also necessary for empathy for the self [2]. The cognitive capacities underlying empathy for the self (e.g. self-awareness and self-reflection) emerge in early childhood and continue developing through adolescence [2,6]. Compared with empathy for others, empathy for the self has received less empirical study. Below, we focus on self-compassion as a core kind, self-oriented emotion.

#### (i) Development of self-compassion

Self-compassion is a positively valenced, self-oriented emotion characterized by a gentle, non-judgmental stance towards the self in times of pain or failure and is conceptualized to include self-kindness, mindfulness and a relational awareness of common humanity [46]. Research on the development of self-compassion has focused on adolescents (for a review see [34]). Longitudinal studies suggest modest stability in self-compassion across middle childhood [47] and early adolescence [22]. Cross-sectional studies with adolescents have yielded more mixed findings. Some show no association between age and self-compassion [48,49], while others suggest an interaction between age and gender, with older adolescent girls reporting the lowest levels compared with boys and younger adolescent girls [50]. Cross-sectional work spanning adolescence and adulthood generally indicates a positive association between age and self-compassion [51,52], though one study found a reverse U-shaped pattern, with self-compassion peaking at age 77, and then declining [53].

### (c) Development of gratitude

Gratitude is a positively valenced, self-oriented emotion that arises when one recognizes and feels appreciation for a benefit provided by another [15]. Early signs of gratitude appear in toddlerhood, with children being able to recognize the intentionality behind others' helpful acts, and beginning to respond prosocially based on gratitude-based motivations [54,55]. For instance, 2-year-olds who are helped by another person show greater internal motivation (indexed by pupil dilation) to help their benefactor compared with someone who has not helped them [54].

With age, expressions of gratitude become more frequent and sophisticated, developing in parallel with children's advancing knowledge of emotions and mental states. For example, 4-year-olds (but not 3-year-olds) who are helped by someone are more likely to share with another child, and their positive emotions towards their benefactor predict their subsequent prosocial behaviour [56]. Longitudinal evidence shows that emotion and mental state knowledge at ages 3–4 years predicts greater knowledge of gratitude at the age of 5 years [57]. School-age children also show developmental shifts in how they conceptualize gratitude; 7–13-year-olds provide increasingly abstract definitions and report feeling grateful for more abstract benefits [58]. Similarly, while children of ages 7–14 years commonly express gratitude, older children are more likely to reciprocate with something of clear value to the benefactor, reflecting a more sophisticated form of gratitude [59,60]. Thus, gratitude emerges early and becomes more complex as children's self- and other-oriented emotional and cognitive capacities grow.

## 4. Mechanisms and functions of caring environments in shaping kind emotions

**Figure 1.**
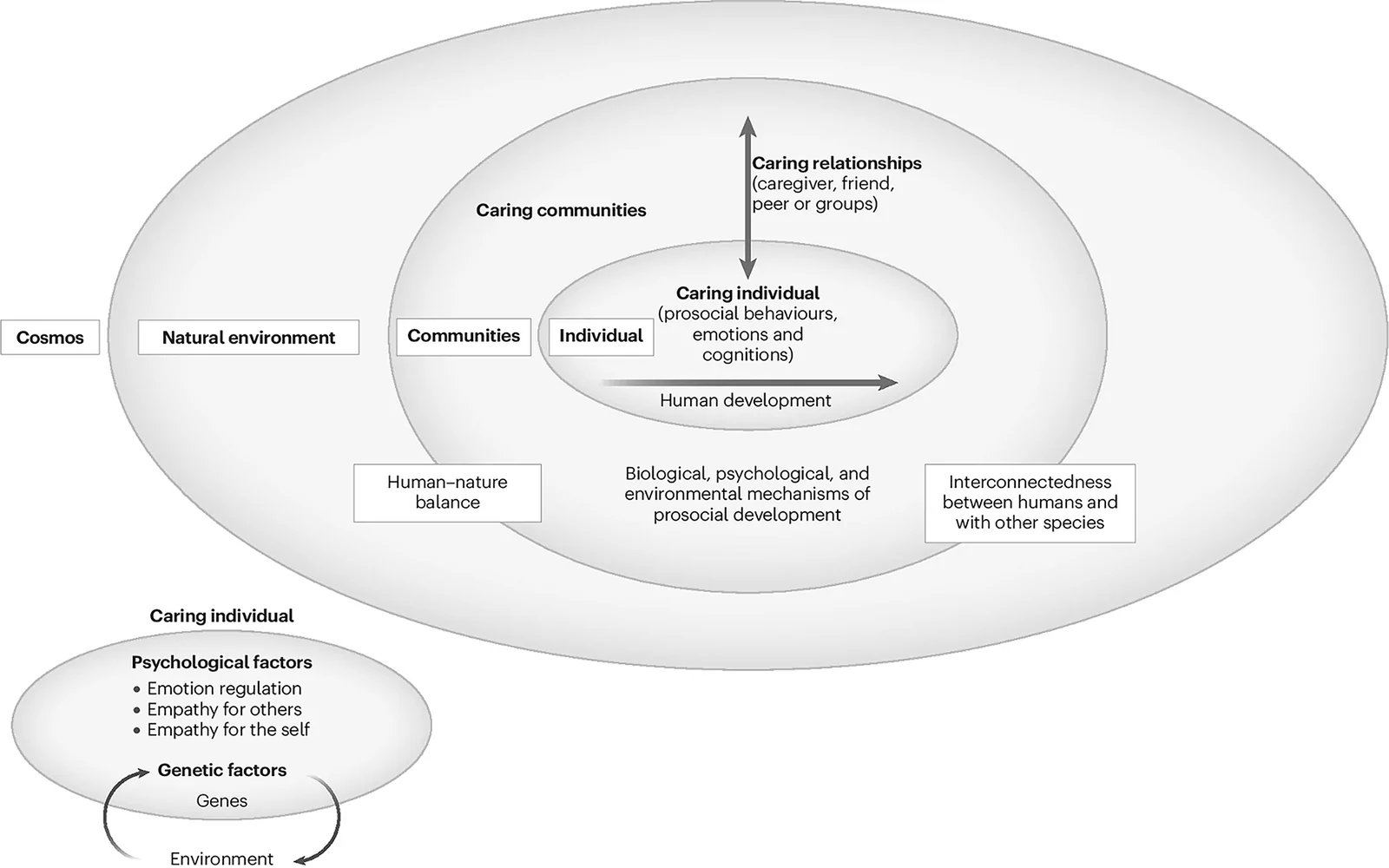
The web of care [2]. Note: permission from Springer Nature.

We use the term *caring environments* to refer to features of environmental contexts (i.e. parenting practices, familial and peer relationships, educational settings and community factors) that support the development of kind emotions, often involving relational processes and interactions characterized by warmth, sensitivity and support. As children grow, numerous environmental factors are related to, and predict, kind emotions. Malti & Speidel [2] developed the *web of care* as a meta-conceptual framework that builds upon developmental-relational theory to conceptualize prosociality as an individual characteristic that unfolds within and through a series of nested, caring relationships and processes (i.e. environments). These relationships involve ongoing interactions between humans, non-humans, the natural environment and the cosmos. As illustrated in figure 1, the web of care frames caring relationships and environments as directly and indirectly shaping children's development of kindness and caring. These nested environments include close, caring relationships within familial, peer and friendship settings, as well as more broadly within educational, cultural and community settings. These caring relationships are embedded within the broader natural world, which includes children's everyday interactions with animals, plants, trees, rivers, spaces and other living and non-living entities. Finally, these interconnected networks are all part of the larger cosmos. As is illustrated in figure 1, these relationships are bidirectional in that environmental factors and individual factors influence one another. Furthermore, multiple environmental factors may interact with one another to influence kind emotions [61].

Next, we apply the web of care to examine various caring environments associated with the development of kind emotions, focusing on (i) caring family environments, (ii) caring peer relationships and friendships, (iii) caring school environments, and (iv) caring communities. Across these factors, several key themes emerge. First, kind emotions are nurtured within complex, multi-level environments, reflecting ongoing interplay and interactions between children, families, peers, schools, communities and natural settings. Second, bidirectional processes are common whereby children influence and are influenced by their caring environments. Third, certain features of caring environments seem to be consistently supportive across contexts, including warmth, sensitivity, inclusion, safety, structured support and opportunities for perspective-taking. Amidst these commonalities, the web of care is intended to function as a flexible framework which acknowledges that child-rearing practices, social norms and pathways to kind emotions may vary across different cultural contexts, developmental stages and social contexts (e.g. experiences of adversity). Although a comprehensive review of cross-cultural research is beyond the scope of this paper, our focus is on processes that appear to be relatively robust across contexts, while acknowledging that variation between cultures and settings is common and important for understanding individual differences in prosocial behaviours.

## 5. Caring family environments

Numerous theoretical frameworks emphasize the importance of positive early familial interactions in supporting children's moral and prosocial development [62,63], including the development of empathy [31]. In this section, we focus on caring family relationships as they relate to kind emotions, including caring parent behaviours, caring child behaviours and caring sibling behaviours.

### (a) Caring parent behaviours

Caring parent–child relationships play a central role in shaping children's kind emotions, including empathy for others [64,65], empathy for the self (e.g. self-compassion) [66] and gratitude [67]. Attachment theory [68–71] offers a relational framework for understanding these processes, highlighting how sensitive, responsive caring fosters secure attachment, which, in turn, supports better emotion regulation, healthy relational schemas and supportive physiological responses that nurture the capacity to care for others [72–74] and the self [66].

Building on this theoretical work, growing research has focused on positive parenting as a more practice-oriented approach for understanding the development of children's positive social-emotional capacities, including kind emotions. Positive parenting is an umbrella construct encompassing a range of beneficial parenting practices [75]. It includes supportive, warm, responsive and sensitive caring that fosters positive aspects of children's social-emotional development [76–78]. Multiple studies have documented positive associations between various dimensions of positive parenting (i.e. parental sensitivity, parent–child synchrony, emotion-related socialization and warmth) and children's empathy for others.

Maternal sensitivity (i.e. the ability to accurately perceive and respond to children's cues [79]) has been identified as a key factor fostering empathy for others [80–82]. Similarly, in the first year of life, parent–child synchrony (i.e. coordinated exchanges between parent and child) predicts empathy for others later in childhood and adolescence [83–85]. Another factor is parents' emotion-related socialization behaviours (i.e. modelling, validation and discussion of emotions), which provide children opportunities to learn how to understand and respond to others' emotions, and have been associated with children and adolescents' empathic concern towards others [81,86], self-compassion [87] and gratitude [67,88]. Finally, parental warmth (i.e. positive affect, affection and enjoyment of the child [89]) has been linked to children's development of kind emotions in early [90] and later childhood [91,92]. There is also empirical evidence linking warm parenting to empathy for the self in adolescent samples. For example, using a cross-sectional design, Ren *et al.* [93] found that the positive association between warm parenting and adolescent mental health is mediated by higher self-kindness in early, middle, and late adolescent samples. Similarly, Dakers & Guse [94] reported that among South African adolescents, parental warmth is positively associated with self-compassion. Parental warmth is also associated with the development of gratitude in adolescents; specifically, parental warmth is associated with higher subsequent gratitude through friendship quality [95].

Theoretical [96] and empirical research [29,37–39] suggest that some caring interactions may contribute more strongly, or in different ways, than others. For instance, Davidov & Grusec [89] found that maternal responsiveness to distress (but not general maternal warmth) predicted children's empathy for others, whereas warmth predicted better regulation of positive affect in early school-age children. A similar pattern emerges in infancy; maternal responsiveness to infants' distress at three and six months predicts higher empathy towards others at 12 and 18 months, while other dimensions of sensitive parenting do not [97].

The findings highlight the importance of considering various aspects of parenting, as specific parenting behaviours may contribute uniquely to kind emotions [98].

### (b) Caring child behaviours

In addition to parents' behaviours, children play an active role in shaping caring parent–child relationships [99]. Children's responsiveness to parental socialization (including efforts to foster empathy for others) can vary based on individual characteristics, such as temperament [100]. For example, Wagers and colleagues demonstrated in a longitudinal study of toddlers and their mothers that parental warmth predicted higher empathy among children with low inhibition, whereas maternal reasoning was associated with lower empathy among children with high inhibition [101]. Furthermore, Partington & Hastings [75] highlighted the bidirectional nature of parental care and kind emotions in children. Maternal warmth and reasoning were not only predictive of children's subsequent concern for others but were also shaped by children's own levels of concern over time. For instance, boys who displayed greater concern appeared to elicit reductions in maternal reasoning, perhaps because their mothers perceived them as already being emotionally attuned [96].

Emerging evidence suggests that children's self-compassion may also show bidirectional links with parenting. Using a longitudinal design, Song *et al.* [102] found that children's self-compassion in middle childhood is linked to the quality of parent–child attachment; secure attachment fosters children's self-compassion, while children with higher self-compassion have higher-quality parent–child relationships. Similarly, Yeung [103] reported that positive family functioning is predictive of higher gratitude and lower depression among parents and children and that children's and parents' gratitude and depression affect each other bidirectionally. This reciprocal dynamic has the potential to create a reinforcing spiral effect, whereby supportive interactions and children's self-compassion mutually strengthen one another across development [102].

These findings underscore that children are not passive recipients of care, but rather active contributors to the creation and maintenance of caring parent–child relationships.

### (c) Caring sibling relationships

Sibling relationships offer a unique context for moral development (including kindness), given that they provide frequent opportunities for emotional exchange, conflict resolution and perspective-taking [104,105].

Positive sibling relationships offer added value to children's kind emotions above and beyond caring parent–child relationships [106]. For example, sibling affection predicts higher subsequent sympathy in adolescents [106]. Other studies indicate that older siblings may play an important role in fostering sympathy in younger siblings [107]. Extending this work, Jambon *et al.* [108] found reciprocal effects of older sibling–younger sibling empathy over 18 months. Similarly, sibling warmth is positively associated with empathy, whereas sibling conflict is associated with lower empathy. Importantly, these associations seem to strengthen with age, suggesting that siblings increasingly shape each other's empathic development, contributing to the broader caring climate of the family [109].

## 6. Caring peer relationships and caring friendships

Children form peer relationships and friendships from an early age [110]. Early interactions with other children provide a platform for children to experiment with different behaviours, observe consequences and develop an understanding of their own and others' behaviours and emotions in close relationships with others who are similar to themselves [111]. Peer relationships and friendships are distinct, but both pose important influences on the development of kind emotions [110].

### (a) Caring peer relationships

High-quality peer relationships are marked by warmth, support, respect, cooperation and constructive conflict resolution, and are linked to the development of empathy for others and the self [105,106]. In a meta-analytic review, adolescents with higher-quality peer relationships show higher emotional and cognitive empathy for others [112]. Importantly, there is evidence that this link is reciprocal; empathy for others is associated with better peer relationship quality by increasing prosocial behaviour and constructive conflict management [113–115]. Vice versa, empathy for others tends to increase over time in higher-quality peer relationships and decrease over time in lower-quality peer relationships [115,116]. There is relatively less literature examining the link between peer relationship quality and empathy for the self, but existing work suggests similar dynamics for self-compassion [117].

### (b) Caring friendships

Friendships are more intimate, sensitive and voluntary than peer relationships and are characterized by mutual affection, goodwill, trust and respect [110]. Given that they provide a context of support, security and affection, friendships are considered an important caring environment that facilitates the development of kind emotions [118,119]. In fact, the association between close friendships and empathy for others and the self is well-established; higher empathy supports the formation of high-quality friendships, and empathy develops and deepens within these relationships through emotion socialization and supportive, reciprocal interactions [120–123]. This bidirectional relationship between friendship quality and empathy has been confirmed in longitudinal work [115]. Notably, friendships are not always supportive for the development of kindness. For example, children who are aggressive tend to form friendships with other aggressive peers, which can reinforce and escalate aggressive behaviour [110,124].

## 7. Caring educational environments

Throughout childhood and adolescence, educational environments become increasingly important to children's development of kindness, as these environments progressively take on functions previously associated with the family, such as attachment, care and belonging [125,126]. In this section, we focus on three aspects of educational environments that are expected to shape kind emotions: formal education, school climate and student–teacher relationships [127].

### (a) Caring in the context of formal education

Contemporary school systems still closely resemble their industrial-age origins, when mass education emerged to efficiently and uniformly shape children into functional adults who can meet the demands of an increasingly commercialized, competitive and complex society [128]. Within these environments, children are introduced to a system of norms, roles, expectations, assessments, rewards and punishments, designed to maximize performance within a standardized curriculum. Some have raised concerns about potential negative impacts of such environments on moral development, including prosociality and kindness [129,130]. In fact, empirical studies on medical education suggest that formal schooling may be associated with a decline in empathy for others [131]. By contrast, children in Indigenous communities, whose education is rooted in rich, informal learning experiences, often show more prosocial behaviour than children with extensive experiences of formal schooling [132,133]. Although it remains unclear whether these findings can be generalized to other educational settings, there are some indications that declines in empathy for others may be linked to features of formal educational systems, including time pressure, competition and focus on performance. Competitive environments that foster social comparison can actively suppress empathy and give rise to negative emotions such as envy and schadenfreude [134].

At the same time, schools can also provide powerful, structured platforms to reach and support more children's prosociality, and interest has grown in using these environments to promote children's potential for kindness through social-emotional learning (SEL) programmes. Compelling evidence suggests that empathy for others and the self can be effectively promoted, with positive ripple effects on classroom climate and learning [135–138]. Many nations now embed empathy-related teaching practices into their curricula [139–142]. Importantly, evidence suggests that the effectiveness of SEL depends less on content than on the *quality* of the social interactions they provide, the social-emotional competencies of the teachers implementing them and the extent to which the concepts can be integrated into existing structures and activities [136,143–146]. Group collaboration and opening space for dialogue have the highest potential to foster empathy among students, especially when they enable interactions between groups of diverse backgrounds [145, 147, 148]. Role-play, theatre, storytelling, exercises in perspective-taking, self-reflection or mindfulness, cross-age tutoring, exchanging disclosures of upset feelings and humane experiences with animals can be effective approaches to foster empathy in educational environments [147, 149–152]. Virtual training approaches focusing on promoting empathy for others in students are also on the rise [152–154].

### (b) Caring school climates

School climates are shaped through instructional practices, interactions between children, teachers, peers, classroom organization and the broader built environment, including how spaces and the school building are configured [127,144,155]. Empathy for others thrives when children's emotional needs are met and when they are encouraged to observe and engage in sensitive, responsive interactions [156]. Thus, the presence of a caring school environment that is characterized by safe, supportive and inclusive relationships is considered important for the development of kind emotions [157]. Relatedly, when children engage in kind interactions with others, they help foster a setting where others feel seen, heard and supported. It is this relatedness, the experience of care and the responsibility of caring for others, that fundamentally motivates kind emotions and behaviours that sustain caring relationships [158].

Perceptions of supportive, organized and culturally diverse school environments are associated with higher empathy and social belonging in students [155,159,160,161 ]. Similarly, school climate and classroom management are associated with group dynamics. For example, when peer empathy is high, students are more likely to defend victims in bullying situations [162,163]. These processes may be most effectively achieved when they involve creating interactive spaces that engage all students, staff, families and communities in egalitarian participation, as part of a ‘whole-school approach’ [164]. Inclusive school environments can also help overcome selectiveness in friendships (a phenomenon called ‘homophily’), contributing to the formation of richer friendship networks among children [165]. This work aligns with well-known approaches in moral education, such as Kohlberg's just community approach, which emphasizes creating supportive environments where students and teachers jointly contribute to making decisions and resolving conflicts, with the goal of supporting students' moral development through respect for the rights and needs of others [166]. Empathy towards peers can also be intentionally fostered through school-wide peer support and mentoring programmes, which have demonstrated effectiveness in reducing bullying while promoting well-being and academic success [167].

### (c) Caring student–teacher relationships

Caring student–teacher relationships are a pivotal component of caring school environments [155]. Evidence across age groups consistently links caring student–teacher relationships with higher empathy and prosocial behaviour in the classroom, sometimes exceeding the influence of core family relationships [72,168,169]. These associations also appear to be bidirectional; warm, supportive student–teacher interactions foster empathy and prosocial behaviour, and in turn higher empathy in students strengthens caring relationships with teachers [168,170]. As children transition into secondary school, psychological closeness between students and teachers tends to decline, but teachers remain central figures in children's lives as mentors, role models and secure bases [144,171,172]. While much of the research in this topic has focused on empathy for others, emerging evidence suggests that gratitude within student–teacher relationships is associated with higher child well-being and reduced teacher stress and burnout [173,174]. This is important given that rising academic and emotional demands placed on teachers can contribute to teacher burnout, which can erode empathy and diminish the quality of student–teacher relationships [144].

## 8. Caring communities and the natural environment

Moving beyond familial, peer and educational contexts, broader caring communities (e.g. neighbourhoods, racial/ethnic communities, wider relational networks, cities and natural environments) can also contribute to the development of kind emotions. As these factors are often more distal, their impacts can be harder to evaluate. Indeed, there is limited research examining links between community environments, societal factors and natural environments and children's kind emotions. Nevertheless, existing evidence provides some insights into how caring community-based experiences, social connections and broader environmental contexts are linked to children's kind emotions.

One area of research at the community level examines efforts to promote positive community change by supporting young people's capacity for empathy through service engagement, service-learning and empathy-focused initiatives. Such efforts aim to nurture children's understanding of community needs, cultivate a sense of responsibility and care towards others and build their sense of efficacy in their ability to positively impact others. Empirical evidence suggests benefits of such initiatives on kind emotions and related prosocial behaviours and attitudes. For example, a study of elementary school-aged children participating in a service-learning programme found increases in their empathy and community engagement following the programme [175]. Similarly, a qualitative study with teachers of preschool-aged children found that an empathy-focused training improves teachers' capacity to promote empathy, which, in turn, supports children's empathy towards animals and nature [176]. Interventions targeting children's empathy also show promise in promoting kinder community environments for peers who have experienced adversity. For example, an empathy-based intervention for 8- to 11-year-old host children was successful in increasing their empathy and helping intentions toward refugee peers [177].

Research also examines how existing community conditions, such as a sense of belonging and community cohesion, are related to children's kindness. In one study using a large, nationally representative sample of preschool-aged children in Australia, sense of neighbourhood belonging (indexed by carers' trust in neighbours and sense of identity within their neighbourhood) was positively associated with children's prosocial behaviour [178]. Conversely, harmful community factors can act to undermine empathy. For example, exposure to community violence is associated with lower empathy among adolescents with pre-existing aggressive behaviours [179]. Other research suggests that kindness can survive and even thrive in contexts of adversity, including among adolescents exposed to war-related violence [180,181], and racial discrimination [182]. Theoretical perspectives underlying these conflicting patterns of findings suggest that in some cases, experiences of severe adversity may result in ‘altruism born of suffering’ whereby children's experiences of extreme pain and adversity make them more sensitive to the distress of others, and thus, more empathetic and motivated to support others [2,183].

 Beyond social and structural factors, there is emerging evidence that the natural environment is related to the development of kind emotions and prosociality. For instance, children show increases in their prosocial behaviour following an outdoor play intervention [184]. Similarly, a recent systematic review of studies with children of ages 2–7 years in early childhood education settings found that outdoor play in natural environments is associated with positive developmental outcomes tied to kind emotions, including better social capacities and prosocial behaviour [185]. School-aged children's caring interactions with animals are also associated with higher empathy and prosocial behaviour [186,187]. Despite these promising findings, formal evaluations of links between caring natural environments and kind emotions are very limited, and rarely include specific indicators of kind emotions.

## 9. Synthesis and future directions

This review highlights that kind emotions emerge early and continue to develop as children's cognitive capacities and social interactions grow increasingly complex, spanning the different relational mechanisms highlighted in the web of care framework. Notably, across environmental domains, relationships characterized by warmth, responsivity, sensitivity and structure generally support children's empathy for others, empathy for the self and gratitude. It is possible that these relational qualities function through common psychological and developmental mechanisms. For example, relationships marked by warmth, responsivity and sensitivity may foster attachment security, which, in turn, supports children's early social-emotional development, including emerging empathy and the capacity for self-regulation [188]. Meta-analytic evidence seems to corroborate this theoretical perspective, as there are reliable associations between secure attachment (which often characterize relationships high in warmth and sensitivity) and higher empathy in children and adolescents [189]. Caring qualities like warmth, responsivity, and sensitivity may be developmentally potent across contexts because they engage evolutionarily conserved domains of social interaction that evolved to address recurring adaptive challenges in human development, even though specific behaviours through which these qualities are expressed may vary across cultures [96]. Thus, what may be universal is the foundational domains these common qualities activate and the kind emotions these domains reliably support, rather than the specific behavioural forms that these caring qualities take.

In addition, caring that is characterized by structure and developmentally appropriate behavioural control in early relationships may function to promote children's kind emotions through social learning and socialization-based processes. Through repeated exposure to scaffolded emotion-based interactions, children gradually internalize observed patterns of interpreting, regulating and expressing self- and other-oriented emotions, including kind emotions [190]. This process is thought to support the formation of a moral identity and the autonomous self-conscious integration of moral virtues like kindness into one's self-concept [191]. When kindness becomes a central feature of an adolescent's identity, kindness-related mental schemas, including kind emotions, may be more readily activated when appraising social situations [192,193]. Future work should continue to explore the commonalities in caring relationships that promote kind emotions, and their accompanying mechanisms.

Notably, the effects of caring environments can vary depending on individual differences such as age, culture and experiences of adversity. For example, in some cases, experiences of adversity can function to enhance empathy and in other cases can act to undermine it, indicating multi-final developmental pathways [2]. Illustrating this heterogeneity, in a recent scoping review of 43 studies examining links between early life adversity and empathy, 15 studies showed that early adversity was associated with increased empathy, 19 showed that early adversity was associated with decreased empathy and 12 showed a null effect [194]. Despite this variability, there is limited work systematically unpacking the developmental mechanisms that help us understand these multi-final outcomes. However, amidst the research that does exist, individual capacities such as emotion regulation and enhanced empathy, and relational factors like connections to supportive, warm and responsive relationships with carers or in school contexts act as protective factors that promote prosocial outcomes in contexts of adversity [195–197]. More integrative, mechanism-focused research is needed to better understand the factors that explain the complex nature of the association between adversity and kind emotions and prosocial outcomes.

To date, the existing evidence is most robust regarding links between caring environments and empathy for others, while research on empathy for the self and gratitude remains relatively limited, particularly in early childhood. Related to this, measures of self-oriented kind emotions (e.g. self-compassion) are less developed than those for other-oriented emotions, and are especially rare in early childhood [198]. Evidence in the literature also suggests that other- and self-oriented kind emotions are interdependent in that they co-develop and often act to reinforce each other [22,199]. These nuances and gaps highlight the need for future research considering multiple kind emotions simultaneously to disentangle similarities and differences in how caring environments uniquely or collaboratively contribute to other- and self-oriented kind emotions.

A synthesis of the reviewed literature points to several practical implications. First, interventions and services that target more robust environmental and relational links to kind emotions (e.g. warmth, sensitivity, inclusion and structure) may be particularly successful in promoting children's kind emotions, and in turn, prosocial and kind actions. Relatedly, and consistent with the web of care framework, interventions and services that simultaneously target multiple ecological levels (e.g. family, school, community and peer contexts) may have additive effects in fostering children's propensity for kindness. For example, the intervention families and schools together (FAST Track) integrates coordinated supports across multiple levels, including a child component focused on social-emotional capacity building, a parent training component designed to foster consistency and positive discipline, a school supports component designed to promote classroom management and a peer-based component supporting social skills [200]. This exemplifies a comprehensive, holistic approach that targets multiple levels of a child's relational world, aligning with concepts highlighted in the web of care framework. FAST Track was specifically designed for children at risk for conduct disorders and has been tied to long-term reductions in antisocial behaviour and promotion of prosocial outcomes [201], highlighting the value of targeting multiple relational processes across a child's web of care. Tailoring multi-level interventions such as this to children's developmental stage and community context may also help ensure that caring environments are appropriately moulded to the strengths and needs of different children.

The current review also reveals notable gaps and limitations that remain in the literature. For example, although all levels of the web of care are theorized to play an important role in contributing to the development of kind emotions, more research is needed to investigate the role of more distal caring environments (e.g. community, nature and the cosmos and spirituality). In addition, more research is warranted examining cross-cultural comparisons and specificities of relational processes across different levels of the web of care and across different community groups to help inform developmental knowledge and tailored intervention efforts. Finally, much of the existing research on caring environments is correlational and should therefore be interpreted with the appropriate caution. Where possible, experimental and longitudinal research will be beneficial to clarify causal pathways and directionality, including in intervention studies.

## 10. Conclusion

In summary, using the web of care as a meta-theoretical framework, the literature illustrates how caring environments at the familial, educational, societal and natural levels support the development of kind emotions. Synthesizing across these levels provides insight into the processes that nurture empathy, self-compassion and gratitude, including how these emotions are expressed non-verbally through facial expressions, gestures, touch and the functions they serve in promoting kind, prosocial behaviours. Together, such research can deepen our understanding of what is needed to nurture and grow kinder individuals and communities, and ultimately a kinder humanity.

## Data Availability

This article has no additional data.

## Theme

One contribution of 19 to a theme issue ‘Mechanisms, development, phylogeny and functions of emotional expressions’.
